# Schizophrenia Plays a Negative Role in the Pathological Development of Myocardial Infarction at Multiple Biological Levels

**DOI:** 10.3389/fgene.2021.607690

**Published:** 2021-06-03

**Authors:** Xiaorong Yang, Yao Chen, Huiyao Wang, Xia Fu, Kamil Can Kural, Hongbao Cao, Ying Li

**Affiliations:** ^1^Department of Outpatient, West China Hospital, Sichuan University/West China School of Nursing, Sichuan University, Chengdu, China; ^2^Mental Health Center of West China Hospital, Sichuan University, Chengdu, China; ^3^School of Systems Biology, George Mason University (GMU), Fairfax, VA, United States; ^4^Department of Psychiatry, First Hospital/First Clinical Medical College of Shanxi Medical University, Taiyuan, China; ^5^The Center of Gerontology and Geriatrics, National Clinical Research Center for Geriatrics, West China Hospital, Sichuan University, Chengdu, China

**Keywords:** schizophrenia, myocardial infarction, genetic pathway, regression analysis, meta-analysis

## Abstract

It has shown that schizophrenia (SCZ) is associated with a higher chance of myocardial infarction (MI) and increased mortality. However, the underlying mechanism is largely unknown. Here, we first constructed a literature-based genetic pathway linking SCZ and MI, and then we tested the expression levels of the genes involved in the pathway by a meta-analysis using nine gene expression datasets of MI. In addition, a literature-based data mining process was conducted to explore the connection between SCZ at different levels: small molecules, complex molecules, and functional classes. The genetic pathway revealed nine genes connecting SCZ and MI. Specifically, SCZ activates two promoters of MI (IL6 and CRP) and deactivates seven inhibitors of MI (ADIPOQ, SOD2, TXN, NGF, ADORA1, NOS1, and CTNNB1), suggesting that no protective role of SCZ in MI was detected. Meta-analysis showed that one promoter of MI (CRP) presented no significant increase, and six out of seven genetic inhibitors of MI demonstrated minor to moderately increased expression. Therefore, the elevation of CRP and inhibition of the six inhibitors of MI by SCZ could be critical pathways to promote MI. Nine other regulators of MI were influenced by SCZ, including two gene families (inflammatory cytokine and IL1 family), five small molecules (lipid peroxide, superoxide, ATP, ascorbic acid, melatonin, arachidonic acid), and two complexes (CaM kinase 2 and IL23). Our results suggested that SCZ promotes the development and progression of MI at different levels, including genes, small molecules, complex molecules, and functional classes.

## Introduction

Schizophrenia (SCZ) is one of the most chronically disabled mental illnesses (McGrath et al., 2008). The early manifestations of the disease usually appear in middle and late adolescence, and the clinical onset usually begins 2–5 years later. Patients with SCZ pose unique challenges due to affect, cognition, and socio-demographic factors. Myocardial infarction (MI) and afterward heart failure are the significant causes of death and disability in the developed countries, characterized by acute myocardial ischemia derived from coronary artery occlusion, myocardial injury, and even necrosis (Lu et al., 2015; Sakaguchi et al., 2020).

An increasing amount of literature has discussed the strong correlations between mental disorders and increased MI mortality, especially in patients with SCZ (Nielsen et al., 2015). A study using a nationwide inpatient sample examines the outcomes of Acute Myocardial Infarction (AMI) in patients with SCZ. They found that 4,648 out of 1,196,698 discharged with AMI were also diagnosed SCZ, and these patients diagnosed with both SCZ and AMI showed higher in-hospital mortality (Karthik et al., 2012). Risks of AMI were raised nearly twofold in younger people with SCZ (age under 35) (Wu et al., 2015). A study by Nielsen et al. (2015) reported that 75% of SCZ patients developed silent MI, which may be related to the psychiatric diseases covering up cardiovascular diseases (Kugathasan et al., 2018). Thus, some studies indicate that SCZ is a significant risk factor of in-hospital mortality in MI patients (Sohn et al., 2015; Wu et al., 2015). Also, high mortality following incident MI in individuals with SCZ may associate with low access to care (Kurdyak et al., 2012).

Despite the clinical outcomes that support the relationship between MI and SCZ (Karthik et al., 2012; Nielsen et al., 2015), the underlying mechanisms of the promotion effect of SCZ on MI are largely unknown. It has been suggested that the pathogenesis of a disease can be explained through a multiscale interactome network of proteins, drug targets, and biological functions (Ruiz et al., 2021), and the network-based location of each disease module determines its pathological relationship to other diseases (Menche et al., 2015). Here, we studied the potential influence of SCZ on MI at different levels (genes, small molecules, complex molecules, and functional classes), with functional pathways constructed. Moreover, a meta-analysis was conducted using MI expression data to explore the gene expression variation within the SCZ-driven MI-regulating genetic pathway. Results from this study may add new insights into the understanding of the negative roles that SCZ plays in the pathological development of MI, which is critical in the prevention and treatment of MI in SCZ patients.

## Materials and Methods

This study is organized as follows. First, we conducted a Natural Language Processing (NLP)-based literature data-mining (Daraselia et al., 2004) to construct a genetic pathway connecting SCZ and MI. Second, we performed a meta-analysis to test the gene expression variations of the pathway genes in MI patients. Lastly, we explored SCZ-driven MI regulators at other levels, including small molecules, functional gene classes, and complex molecules.

### Identify SCZ-MI Genetic Pathways

Assisted by Pathway Studio (^1^version 12.3), we conducted an NLP-based large-scale literature data mining to identify common genes that were downstream targets of SCZ and up-regulators of MI. That is, each gene was identified as influenced by SCZ, and was also regulating MI, forming a SCZ→Gene→MI relationship. For each relationship identified, there were at least three independent supporting references, which were provided in the supplementary material SCZ_MI→Ref4GeneticPathway, including the title, DOI/PMID, and the sentences where the relationship was identified. The process was conducted by using MedScan (Daraselia et al., 2004), an NLP-based literature data-mining tool. The data mining covered over 24 million PubMed abstracts and 3.5 million Elsevier and 3rd part full-text papers. Each relationship/edge was built based on the fact extracted from the literature by NLP technology with at least three supporting references. A manually quality control process was enforced to remove unreliable relationships and relationships with non-specific polarities. Here, unreliable relationships refer to these with unmatched sentences, which were false positives by the NLP technique.

All the entities within the remaining network were tested using a meta-analysis with nine independent MI RNA-expression datasets. The purpose of the meta-analysis was to explore the gene expression patterns of these SCZ-drive genes, which may help to understand the literature-based relationships identified. To note, instead of using reported results from original data-related studies, we used the original data to calculate the expression levels. The process is described as follows.

### Selection of Gene Expression Datasets for Meta-Analysis

The MI expression datasets were identified within the GEO database^2^ (Clough and Barrett, 2016). The search was conducted using the keyword “myocardial infarction” with 12,193 items identified. Among these items, 678 studies with series data were selected. We made an outline of the metadata of the identified datasets and selected a sub-set for the meta-analysis with the following steps and criteria: (1) The dataset was array expression data (296 datasets); (2) The original data and the corresponding format file were downloadable (152 datasets; metadata summary of these datasets were presented in Supplementary data SCZ_MIMI_datasets); (3) The model organism of the study was indicated as “human” or “Homo sapiens” (143 datasets); (4) The study design was MI cases vs. healthy control (9 datasets). For step 4, we manually checked the metadata of the 143 datasets from step 3, and the qualified datasets were included for meta-analysis. The nine datasets that satisfied the above criteria were included in the meta-analysis, as shown in Table 1.

**TABLE 1:** The nine myocardial infarction expression datasets selected for meta-analysis.

Dataset GEO ID	#Control	#Case	Country	Study Age	Sample Organism
GSE24519	4	34	Italy	3	Homo sapiens
GSE24591	4	34	Italy	3	Homo sapiens
GSE34198	48	49	Czechia Republic	6	Homo sapiens
GSE48060	21	31	United States	6	Homo sapiens
GSE60993	7	10	South Korea	5	Homo sapiens
GSE60993	7	17	South Korea	5	Homo sapiens
GSE62646	14	84	Poland	6	Homo sapiens
GSE66360	50	49	United States	5	Homo sapiens
GSE97320	3	3	China	3	Homo sapiens

### Meta-Analysis Models

For each gene, the meta-analysis estimated the effect size in terms of gene expression log2 fold-change (LFC). Results from using both the random-effects model and fixed-effect model were compared following the statistics estimation used by Borenstein et al. (2010). To determine the heterogeneity of the datasets, between- and within-study variance was calculated and compared. When the total variance (Cochran’s Q statistic) was no bigger than the expected value of the between-study variances (df), the model sets the ISq (percentage of the within- over between-study variance) to zero. In this case, the fixed-effect model, instead of the random-effects model, will be selected for the meta-analysis. The definition of Cochran’s Q statistic, df, and ISq was provided in Eq. (1) to (3) (Borenstein et al., 2010). All analyses were performed using Matlab (R2017a version).

(1) Q=∑i=1kWi⁢Ti2-(∑i=1kWi⁢Ti)2∑i=1kWi,

Where *T_i_* is the deviation of each study, *W_i_* is the inverse variance of each study, and *k* is the total number of studies.

(2) d⁢f=k-1,

Where k is the total number of studies.

(3) I⁢S⁢q=(Q-d⁢f)/Q,

Where Q is the total variance defined by Equation (1), and *df* is the degree of freedom defined in Equation (2).

### Analysis of Influential Factors

To estimate the possible influence of several factors (e.g., study date, country of origin, and sample size) on the gene expression in MI patients, we conducted a multiple linear regression (MLR) analysis and reported the *P*-values for each of these factors.

### Identification of Additional SCZ-Driven MI Regulators

To explore SCZ-driven MI regulators at other levels, we conducted another NLP-based literature data mining assisted by the network building module of Pathway Studio^3^. The analysis was first performed to identify functional gene class, small molecule, complex molecules, and cells that induced MI’s pathological development, and then these types of entities regulated by SCZ were also identified. The overlapped entities were used to construct SCZ→MI network. To ensure high confidence in the identified relationship, we used the confidence level of three (identified entities were supported by at least three references) to filter the relationships. We presented the identified entities and the corresponding relationships in SCZ_MI→Ref4SMPathway and SCZ_MI→Ref4CellPathway.

## Results

### SCZ-MI Genetic Pathway

**FIGURE 1:**
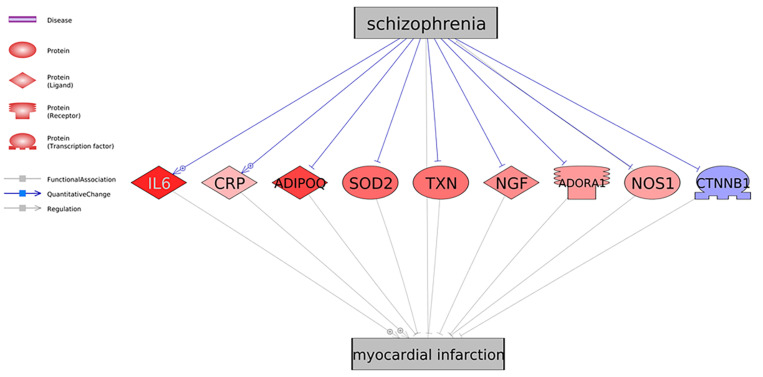
Schizophrenia driven genetic regulators of myocardial infarction. Each relationship was supported by three or more references. The network contains four different types of proteins and three different types of relationships (edges). Genes in red represent an increased gene expression level in myocardial infarction, and blue means decreased.

As shown in Figure 1, there were nine genes driven by SCZ that were MI regulators. Specifically, SCZ activates two MI promoters (IL6 and CRP) and deactivates seven MI inhibitors (ADIPOQ, SOD2, TXN, NGF, ADORA1, NOS1, and CTNNB1). These could be the potential pathways where SCZ plays an essential role in the pathological development and progression of MI. Notably, seven out of the nine genes were MI inhibitors, indicating that SCZ is strongly associated with MI’s pathological development more through MI-inhibitors’ deactivation than through its promoters’ activation. SCZ may play more roles in the progression deterioration than in the initiation of MI. For the details of the pathways presented in Figure 1, please refer to SCZ_MI→Ref4GeneticPathway. From this genetic pathway analysis, we identified no “good” effect of SCZ in MI.

### Meta-Analysis Results

We conducted a meta-analysis using nine MI-expression datasets to test the expression variation of the genes involved in SCZ-driven genetic pathways for MI. We presented the major results of the meta-analysis and MLR analysis in Table 2. The detailed results of the meta-analysis were presented in SCZ_MI→Meta-analysis.

**TABLE 2:** Meta-analysis and Multiple Linear Regression analysis results.

Gene Name	Meta-analysis results				Multiple Linear Regression analysis results (*p*-value)		
	Random-Effects Model (yes = 1; no = 0)	# of Study	Effect size (LFC)	*p*-value	# of Sample	Country	Study Age
ADIPOQ	1	8	0.44	0.10	0.39	*0.013*	0.31
ADORA1	1	9	*0.055*	0.36	0.98	*0.008*	0.06
CRP	0	8	*0.018*	0.40	0.76	0.104	0.53
CTNNB1	0	9	−0.070	0.10	0.88	0.053	0.29
IL6	1	5	0.71	*0.02*	1.00	*3 e*^–^*^5^*	*9 e*^–^*^5^*
NGF	1	3	0.086	0.48	*3 e*^–^*^16^*	*3 e*^–^*^16^*	1.00
NOS1	0	8	*0.044*	0.20	0.83	*0.008*	0.19
SOD2	1	9	0.21	0.24	0.56	*0.087*	0.84
TXN	1	8	0.17	0.34	*6 e*^–^*^6^*	*8 e*^–^*^6^*	1.00

As shown in Table 2, population region (Country) was suggested as a significant influential factor for the expression of almost all the genes tested except CRP and CTNNB1. Patients from different countries usually carry racial and ethnic variations that influence gene expression patterns (Hicks et al., 2013). While the sample size only influences the expression of NGF and TXN (*p*-value = 3e^–16^ and 6e^–6^, respectively), and study age seems to be an influential factor for IL6 alone (*p*-value = 9e^–5^). These results suggested the complexity of the disease of MI, which could be influenced by multiple factors.

As shown in Figure 1 and Table 2, only one MI promoter (IL6) was significantly up-regulated in MI patients (LFC = 0.71, *p*-value = 0.021). The other MI promoter (CRP) presented no significant expression change (LFC = 0.018; *p*-value = 0.40). Therefore, the activation of CRP could be a required course where SCZ promotes MI’s pathological development.

We identified two inhibitors of MI, which got moderate elevation in their expression levels, including ADIPOQ and SOD2 (LFC = 0.44 and 0.21, respectively; *p*-value = 0.10 and 0.24, respectively). Therefore, inhibiting these two genes’ activity could be another path that SCZ contributes to the promotion of MI. Most of the other MI inhibitors demonstrated minor elevated expression except CTNNB1 (LFC = −0.069). The increased expressions of all these inhibitors of MI were beneficial in the progression MI. Thus, by deactivating these MI inhibitors, SCZ could worsen the progression of MI.

### SCZ-Driven Small Molecule Regulators of MI

**FIGURE 2:**
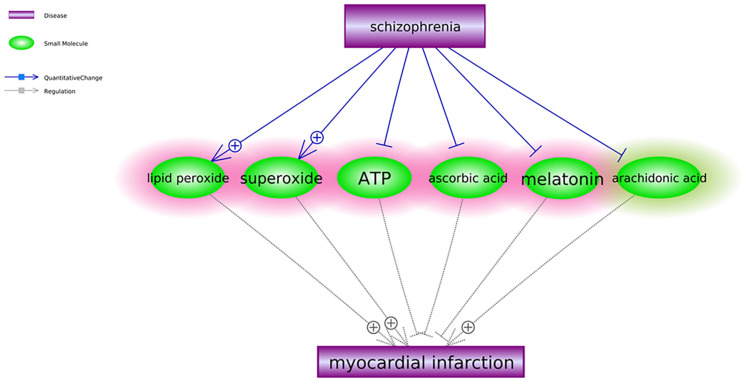
Schizophrenia-driven small molecular regulators of myocardial infarction. Each relationship was supported by three or more references. The network contains two different types of relationships (edges): quantitative change and regulation. The small molecules highlighted in red were the regulators of myocardial infarction driven by schizophrenia to play antagonistic roles in the development and progression of myocardial infarction. The ones in green play antagonistic roles in the development and progression of myocardial infarction.

To explore the connections between SCZ and MI in other levels, we first identified the small molecules that were downstream targets of SCZ and upstream regulators of MI. Three or more references supported each of these relationships (see SCZ_MIRef4SMPathways). Six small molecules satisfied our data mining criteria and formed the small molecule pathway, as shown in Figure 2. Among these six small molecules, SCZ activates two out of three MI promoters and deactivates all three MI inhibitors. Although SCZ could also deactivate one MI promoter (arachidonic acid), the overall conclusion from the small molecular pathway (Figure 2) is consistent with the genetic pathway (Figure 1)—SCZ plays a more negative than positive role at a small molecular level in the pathological development of MI.

### SCZ Driven Regulators of MI at Gene Family and Complex Level

**FIGURE 3:**
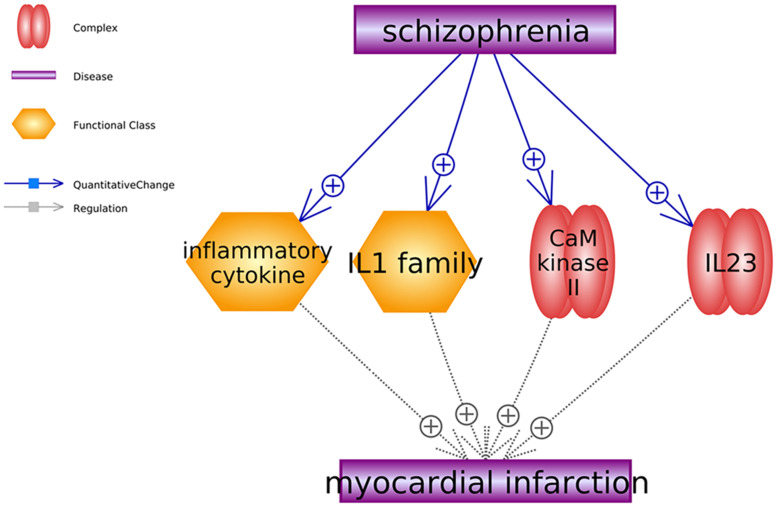
Schizophrenia-driven cells, gene families and complex as regulators of myocardial infarction. Each relationship was supported by three or more references. The network contains two types of relationships (quantitative change and regulation).

Besides small molecules, we also identified two gene families (inflammatory cytokine and IL1 family) and two complexes (CaM kinase 2 and IL23) that were promoters of MI and stimulated by SCZ. To note, the gene family and complex pathways shown in Figure 3 support only the negative influence of SCZ on MI without a positive effect identified. For the details of the pathways presented in Figure 3, please refer to SCZ_MI→Ref4FCPathway.

## Discussion

This study explored the SCZ influenced MI regulators at multiple levels: genetic, gene family, small molecule, and complex. Corresponding pathways were constructed with a meta-analysis to test the gene expression within the genetic pathway in MI patients. The pathway built suggested a negative role of SCZ in MI’s pathological development, which is consistent with previous studies (Karthik et al., 2012; Nielsen et al., 2015). However, different from the clinical research exploring the co-occurrence and common clinical features of two diseases, this study mainly focused on experiment data-based studies to uncover potential mechanisms underlying the clinical association between SCZ and MI.

Firstly, we identified the potential association between SCZ and MI at the genetic level through the connection with nine common genes (Figure 1). Most of the pathways presented in Figure 1 pointed to a negative role of SCZ in the pathological development of MI. For example, the interleukin-6 (IL-6) serum concentrations of SCZ patients was confirmed by multiple studies to get significantly elevated (El Kissi et al., 2015), which was shown to be associated with clinical progression of unstable angina and increased risk of MI (Deten et al., 2003; Gori et al., 2006). Our meta-analysis result confirmed that elevated IL-6 expressions in patients with MI (LFC = 0.71, *p*-value = 0.021). Therefore, the SCZ-IL6-MI could be one of the pathological paths where SCZ promotes MI.

The meta-analysis also showed that the expression of CRP, a promoter of MI, was not significantly elevated in the nine MI-datasets employed in the meta-analysis (Table 2). However, in chronic SCZ patients, the expression levels of CRP could be significantly increased (Meyer et al., 2009), which has been shown to play an essential role in the development of heart failure after MI (Al Aseri et al., 2019). Therefore, the SCZ-CRP-MI pathway could be an essential mechanism explaining the promotion role of SCZ in the progression of MI. Besides the effect of SCZ on the MI promoters, most of the MI inhibitors demonstrated increased expression in MI (LFC > 0), which indicates that SCZ may play more roles in the progression deterioration than in the initiation of MI.

However, we noted that the early stage of SCZ could play some protective role in MI progression through the up-regulation of TXN (an MI inhibitor) (Figure 1). The overexpression of TXN has been suggested as a therapeutical target for MI (Sag et al., 2014; Yang et al., 2016). In the early stage of SCZ, TXN expression could be elevated (Zhang et al., 2009), while in chronic SCZ patients, TXN was shown to be down-regulated, which inverses the role of SCZ back to negative in the progression of MI (Aydın et al., 2018).

ADORA1 forms an oligomeric structure with P2RY1 (Yoshioka et al., 2001) to mediate purine signaling. This activation triggers two different Ca^+2^ release pathways through Calmodulin Kinase 2 (CamKII) and inositol triphosphate (Paredes-Gamero et al., 2006). The release of calcium ions is essential for heart muscle contractions and electrical signal formation. Therefore, an expression increase in ADORA1 might disrupt the intensity of electrical signals generated by heart and muscle tissues. Increased IL-6 expression could be tied to increased release of calcium ions and inositol triphosphate, which causes a positive feedback loop (Bustamante et al., 2014).

Perhaps the most important finding is related to the formation of adherens and gap junction interactions after expression changes of the identified molecules. Deactivation of the beta-catenin transactivating complex is crucial for structural changes in heart muscle formation and maintenance. ADIPOQ, NOS1, TXN, CTNNB1, and CRP play an essential role in the beta-catenin transactivating complex’s deactivation. Beta-catenin is localized at the fascia adherens junction, where it is part of the N-cadherin– actin complex. Over and underexpression of CTNNB1 (beta-catenin) is tied to cardiomyopathies due to structural changes in heart muscle (Sheikh et al., 2009). Beta-catenin is crucial in cell differentiation in the brain as well, and an abnormal Wnt gene expression and plasma protein levels are proven to be related to SCZ in the earlier studies (Hoseth et al., 2018).

We also identified six SCZ-driven small molecules that influence MI’s advance, as shown in Figure 2. Different from the genetic pathway, we also identified one potential “good” pathway (SCZ→arachidonic acid→MI) where SCZ plays a protective role in MI development. It has been shown that arachidonic acid levels are reduced in post mortem and peripheral red blood cell membranes in SCZ (Berger et al., 2016), while the 5-lipoxygenase derivatives of arachidonic acid have been shown to play an important pathogenic role during MI (Lisovyy et al., 2009). However, the regulation of other MI inhibitors and promoters (Figure 2) suggested that SCZ plays a more negative than positive role at the small molecular (compound) level in the pathological development and progression of MI, which is consistent with that of the genetic pathway presented in Figure 1. For instance, SCZ has been shown to reduce the secretion of melatonin, which was implicated as a protector for the cardiac microvascular ischemia to improve the therapeutic outcomes of MI (Zhou et al., 2018; Saberi et al., 2019). More details of the pathway presented in Figure 2 can be found in SCZ_MI→Ref4SMPathway.

Moreover, our study also uncovered four MI promoters (Figure 3), including two SCZ-driven gene families and two complexes. Notably, plasma concentrations of the Interleukin-1 family (IL-1 family) were found significantly increased in SCZ patients (Sirota et al., 2003), which may characteristically modify the process of coronary artery disease associated with Chlamydia pneumonia infection, leading to the development of MI (Momiyama et al., 2001). The expression of calmodulin kinase II (CaM kinase II) was also elevated in the tissues of patients with SCZ (Lee et al., 2010). It has been suggested that CaM kinase II inhibition could improve ventricular functions and restores normal Ca2+ homeostasis after MI (Hund et al., 2008; Fu et al., 2013), while the overexpression of CaM kinase II causes dilated cardiomyopathy and ventricular dysfunction associated with abnormal Ca2+ handling (Hund et al., 2008). Therefore, the pathways presented in Figure 3 may add new insights into the understanding of the negative role of SCZ in MI development.

This study has several limitations that need to be addressed in future work. First, the identification of the SCZ driven MI-regulators was filtered to have support by at least three references. While this decreased the identified entities’ false-positive ratio, some vital information might be lost between SCZ and MI connection, which needs further consideration. Second, the pathways and relations were constructed based on previous studies that were conducted in different backgrounds. Biology experiments are needed to validate any of the relationships identified in this study. Third, the sample size of the MI datasets employed in this study presented significant variance, which influenced the meta-analysis results. Forth, the relationships identified in this study were mainly quantitative changes at the gene/protein expression level. Other types of relation (e.g., genetic change by GWAS study) may add new insights into the understanding of the SCZ-MI relationship. Fifth, due to the limitation of the NLP technique employed in this study, we did not separate different study types (e.g., human, animal, or cell line) when building the pathway given in Figures 1–3.

## Conclusion

We identified 19 SCZ-driven MI-regulators at different biological levels, and SCZ exerts an overall negative influence on MI through the regulation of most of them (18 out of 19). Our results indicated the complexity of the connection between SCZ and MI and may add new insights into the understanding of the negative role that SCZ plays in the pathology of MI.

## Data Availability Statement

Publicly available datasets were analyzed in this study. This data can be found here: https://www.ncbi.nlm.nih.gov/geo/.

## Author Contributions

XY, YC, and YL developed the study design, analyzed the data, and wrote the original manuscript. All authors read and approved the final manuscript.

## Conflict of Interest

The authors declare that the research was conducted in the absence of any commercial or financial relationships that could be construed as a potential conflict of interest.

## Footnotes

1 www.pathwaystudio.com

2 https://www.ncbi.nlm.nih.gov/geo/

3 https://supportcontent.elsevier.com/Support%20Hub/Pathway%20Studio/Network%20Builder%20basic%20_Interactive%20NB%20v114.pdf

## References

[B1] Al Aseri Z. A. Habib S. S. Marzouk A. (2019). Predictive value of high sensitivity C-reactive protein on progression to heart failure occurring after the first myocardial infarction. *Vasc. Health Risk Manag.* 15 221–227. 10.2147/VHRM.S198452 31410012 PMC6643258

[B2] Aydın E. P. Genç A. Dalkıran M. Uyar E. T. Deniz I. Özer ÖA. (2018). Thioredoxin is not a marker for treatment-resistance depression but associated with cognitive function: an rTMS study. *Prog. Neuropsychopharmacol. Biol. Psychiatry* 80 322–328. 10.1016/j.pnpbp.2017.04.025 28442424

[B3] Berger G. E. Smesny S. Schäfer M. R. Milleit B. Langbein K. Hipler U. C. (2016). Niacin skin sensitivity is increased in adolescents at ultra-high risk for psychosis. *PLoS One* 11:e0148429. 10.1371/journal.pone.0148429 26894921 PMC4764507

[B4] Borenstein M. Hedges L. V. Higgins J. P. Rothstein H. R. (2010). A basic introduction to fixed-effect and random-effects models for meta-analysis. *Res. Synth. Methods* 1 97–111. 10.1002/jrsm.12 26061376

[B5] Bustamante M. Fernández-Verdejo R. Jaimovich E. Buvinic S. (2014). Electrical stimulation induces IL-6 in skeletal muscle through extracellular ATP by activating Ca(2+) signals and an IL-6 autocrine loop. *Am. J. Physiol. Endocrinol. Metab.* 306 E869–E882. 10.1152/ajpendo.00450.2013 24518675 PMC3989743

[B6] Clough E. Barrett T. (2016). The gene expression omnibus database. *Methods Mol. Biol.* 1418 93–110. 10.1007/978-1-4939-3578-9_5 27008011 PMC4944384

[B7] Daraselia N. Yuryev A. Egorov S. Novichkova S. Nikitin A. Mazo I. (2004). Extracting human protein interactions from MEDLINE using a full-sentence parser. *Bioinformatics* 20 604–611. 10.1093/bioinformatics/btg452 15033866

[B8] Deten A. Volz H. C. Holzl A. Briest W. Zimmer H. G. (2003). Effect of propranolol on cardiac cytokine expression after myocardial infarction in rats. *Mol. Cell Biochem.* 251 127–137. 14575314

[B9] El Kissi Y. Samoud S. Mtiraoui A. Letaief L. Hannachi N. Ayachi M. (2015). Increased interleukin-17 and decreased BAFF serum levels in drug-free acute schizophrenia. *Psychiatry Res.* 225 58–63. 10.1016/j.psychres.2014.10.007 25453636

[B10] Fu Q. Chen X. Xiang Y. K. (2013). Compartmentalization of β-adrenergic signals in cardiomyocytes. *Trends Cardiovasc. Med.* 23 250–256. 10.1016/j.tcm.2013.02.001 23528751 PMC4264830

[B11] Gori A. M. Sofi F. Corsi A. M. Gazzini A. Sestini I. Lauretani F. (2006). Predictors of vitamin B6 and folate concentrations in older persons: the InCHIANTI study. *Clin. Chem.* 52 1318–1324. 10.1373/clinchem.2005.066217 16690736 PMC2645619

[B12] Hicks C. Miele L. Koganti T. Young-Gaylor L. Rogers D. Vijayakumar V. (2013). Analysis of patterns of gene expression variation within and between ethnic populations in pediatric B-ALL. *Cancer Inform.* 12 155–173. 10.4137/CIN.S11831 24023509 PMC3762614

[B13] Hoseth E. Z. Krull F. Dieset I. Mørch R. H. Hope S. Gardsjord E. S. (2018). Exploring the Wnt signaling pathway in schizophrenia and bipolar disorder. *Transl. Psychiatry* 8:55. 10.1038/s41398-018-0102-1 29507296 PMC5838215

[B14] Hund T. J. Decker K. F. Kanter E. Mohler P. J. Boyden P. A. Schuessler R. B. (2008). Role of activated CaMKII in abnormal calcium homeostasis and I(Na) remodeling after myocardial infarction: insights from mathematical modeling. *J. Mol. Cell Cardiol.* 45 420–428. 10.1016/j.yjmcc.2008.06.007 18639555 PMC2587155

[B15] Karthik M. Gagan K. Abhishek D. Rajesh S. Jawahar M. (2012). Schizophrenia and use of revascularization procedures after acute myocardial infarction. *J. Am. Coll. Cardiol.* 59(Suppl.):E1898. 10.1016/S0735-1097(12)61899-3

[B16] Kugathasan P. Laursen T. M. Grøntved S. Jensen S. E. Aagaard J. Nielsen R. E. (2018). Increased long-term mortality after myocardial infarction in patients with schizophrenia. *Schizophr. Res.* 199 103–108. 10.1016/j.schres.2018.03.015 29555214

[B17] Kurdyak P. Vigod S. Calzavara A. Wodchis W. P. (2012). High mortality and low access to care following incident acute myocardial infarction in individuals with schizophrenia. *Schizophr. Res.* 142 52–57. 10.1016/j.schres.2012.09.003 23021899

[B18] Lee J. G. Cho H. Y. Park S. W. Seo M. K. Kim Y. H. (2010). Effects of olanzapine on brain-derived neurotrophic factor gene promoter activity in SH-SY5Y neuroblastoma cells. *Prog. Neuropsychopharmacol. Biol. Psychiatry* 34 1001–1006. 10.1016/j.pnpbp.2010.05.013 20546816

[B19] Lisovyy O. O. Dosenko V. E. Nagibin V. S. Tumanovska L. V. Korol M. O. Surova O. V. (2009). Cardioprotective effect of 5-lipoxygenase gene (ALOX5) silencing in ischemia-reperfusion. *Acta Biochim. Pol.* 56 687–694. 20011686

[B20] Lu L. Liu M. Sun R. Zheng Y. Zhang P. (2015). Myocardial infarction: symptoms and treatments. *Cell Biochem. Biophys.* 72 865–867. 10.1007/s12013-015-0553-4 25638347

[B21] McGrath J. Saha S. Chant D. Welham J. (2008). Schizophrenia: a concise overview of incidence, prevalence, and mortality. *Epidemiol. Rev.* 30 67–76. 10.1093/epirev/mxn001 18480098

[B22] Menche J. Sharma A. Kitsak M. Ghiassian S. D. Vidal M. Loscalzo J. (2015). Disease networks. Uncovering disease-disease relationships through the incomplete interactome. *Science* 347:1257601. 10.1126/science.1257601 25700523 PMC4435741

[B23] Meyer J. M. McEvoy J. P. Davis V. G. Goff D. C. Nasrallah H. A. Davis S. M. (2009). Inflammatory markers in schizophrenia: comparing antipsychotic effects in phase 1 of the clinical antipsychotic trials of intervention effectiveness study. *Biol. Psychiatry.* 66 1013–1022. 10.1016/j.biopsych.2009.06.005 19640511 PMC3743723

[B24] Momiyama Y. Hirano R. Taniguchi H. Nakamura H. Ohsuzu F. (2001). Effects of interleukin-1 gene polymorphisms on the development of coronary artery disease associated with *Chlamydia pneumoniae* infection. *J. Am. Coll. Cardiol.* 38 712–717. 10.1016/s0735-1097(01)01438-3 11527622

[B25] Nielsen J. Juel J. Alzuhairi K. S. Al Zuhairi K. S. M. Friis R. Graff C. (2015). Unrecognised myocardial infarction in patients with schizophrenia. *Acta Neuropsychiatr.* 27 106–112. 10.1017/neu.2014.41 25582655

[B26] Paredes-Gamero E. J. Craveiro R. B. Pesquero J. B. França J. P. Oshiro M. E. Ferreira A. T. (2006). Activation of P2Y1 receptor triggers two calcium signaling pathways in bone marrow erythroblasts. *Eur. J. Pharmacol.* 534 30–38. 10.1016/j.ejphar.2006.01.010 16487961

[B27] Ruiz C. Zitnik M. Leskovec J. (2021). Identification of disease treatment mechanisms through the multiscale interactome. *Nat. Commun.* 12:1796. 10.1038/s41467-021-21770-8 33741907 PMC7979814

[B28] Saberi K. Pasbakhsh P. Omidi A. Borhani-Haghighi M. Nekoonam S. Omidi N. (2019). Melatonin preconditioning of bone marrow-derived mesenchymal stem cells promotes their engraftment and improves renal regeneration in a rat model of chronic kidney disease. *J. Mol. Hist.* 50 129–140. 10.1007/s10735-019-09812-4 30671880

[B29] Sag C. M. Santos C. X. Shah A. M. (2014). Redox regulation of cardiac hypertrophy. *J. Mol. Cell Cardiol.* 73 103–111. 10.1016/j.yjmcc.2014.02.002 24530760

[B30] Sakaguchi A. Nishiyama C. Kimura W. (2020). Cardiac regeneration as an environmental adaptation. *Biochim. Biophys. Acta Mol. Cell Res.* 1867:118623. 10.1016/j.bbamcr.2019.118623 31837984

[B31] Sheikh F. Ross R. S. Chen J. (2009). Cell-cell connection to cardiac disease. *Trends Cardiovasc. Med.* 19 182–190. 10.1016/j.tcm.2009.12.001 20211433 PMC3601820

[B32] Sirota P. Gavrieli R. Wolach B. (2003). Overproduction of neutrophil radical oxygen species correlates with negative symptoms in schizophrenic patients: parallel studies on neutrophil chemotaxis, superoxide production and bactericidal activity. *Psychiatry Res.* 121 123–132. 10.1016/s0165-1781(03)00222-1 14656447

[B33] Sohn M. Moga D. C. Talbert J. (2015). Mental disorder comorbidity and in-hospital mortality among patients with acute myocardial infarction. *Geriatric. Ment. Health Care* 3 7–11. 10.1016/j.gmhc.2015.04.002

[B34] Wu S. I. Chen S. C. Liu S. I. Sun F. J. Juang J. J. Lee H. C. (2015). Relative risk of acute myocardial infarction in people with schizophrenia and bipolar disorder: a population-based cohort study. *PLoS One* 10:e0134763. 10.1371/journal.pone.0134763 26270347 PMC4536090

[B35] Yang C. J. Yang J. Yang J. Fan Z. X. (2016). Thioredoxin-1 (Trx1) engineered mesenchymal stem cell therapy is a promising feasible therapeutic approach for myocardial infarction. *Int. J. Cardiol.* 206 169–170. 10.1016/j.ijcard.2015.10.150 26527187

[B36] Yoshioka K. Saitoh O. Nakata H. (2001). Heteromeric association creates a P2Y-like adenosine receptor. *Proc. Natl. Acad. Sci. U.S.A.* 98 7617–7622. 10.1073/pnas.121587098 11390975 PMC34717

[B37] Zhang X. Y. Chen D. C. Xiu M. H. Wang F. Qi L. Y. Sun H. Q. (2009). The novel oxidative stress marker thioredoxin is increased in first-episode schizophrenic patients. *Schizophr. Res.* 113 151–157. 10.1016/j.schres.2009.05.016 19540723

[B38] Zhou H. Li D. Zhu P. Ma Q. Toan S. Wang J. (2018). Inhibitory effect of melatonin on necroptosis via repressing the Ripk3-PGAM5-CypD-mPTP pathway attenuates cardiac microvascular ischemia-reperfusion injury. *J. Pineal. Res.* 65:e12503. 10.1111/jpi.12503 29770487

